# Autologous Fibrin Sealants Have Comparable Graft Fixation to an Allogeneic Sealant in a Biomechanical Cadaveric Model of Chondral Defect Repair

**DOI:** 10.1016/j.asmr.2022.03.003

**Published:** 2022-04-15

**Authors:** Benjamin L. Smith, Andrea M. Matuska, Valerie L. Greenwood, Ron Gilat, Coen A. Wijdicks, Brian J. Cole

**Affiliations:** a Department of Orthopedic Research, Arthrex, Inc., Naples, Florida, U.S.A.; b Orthobiologics Research, Arthrex, Inc., Naples, Florida, U.S.A.; c Midwest Orthopaedics at Rush University Medical Center, Chicago, Illinois, U.S.A.; d Department of Orthopaedic Surgery, Shamir Medical Center and Tel Aviv University, Tel Aviv

## Abstract

**Purpose:**

The purpose of this study is to assess the integrity of chondral defect repairs filled with a cartilage allograft and sealed with either allogeneic fibrin sealant or autologous fibrin sealants created with platelet-rich plasma (PRP) or platelet-poor plasma (PPP) in a cadaver model.

**Methods:**

Twenty-millimeter medial femoral condyle (MFC) chondral defects were created in five human cadaveric knees. The defects were filled with particulated cartilage allograft hydrated with PRP from human donors until slightly recessed. Sealants were applied until flush with the articular surface using PRP and autologous thrombin serum, PPP and autologous thrombin serum, or commercial allogeneic sealant. The MFC defects were cycled using a multiaxial testing system to simulate continuous passive motion undergone during rehabilitation. After testing, the repairs were assessed for integrity by quantitatively comparing defect exposure and qualitatively assessing sealant delamination.

**Results:**

The mean defect exposures were 4.20% ± 5.02% for the PRP group, 4.60% ± 5.18% for the PPP group, and 1.80% ± 2.95% for the allogeneic sealant group. No significant differences were observed between groups (*P* = .227), and each group had significantly less defect exposure when compared to the critical clinically relevant value assigned to be 30% (*P* = <.001 for all). No complete sealant delamination was observed, although the allogeneic sealant delaminated with a higher magnitude than did the autologous sealants.

**Conclusions:**

The PRP and PPP sealants were comparable to the allogeneic sealant for graft fixation when used in conjunction with an underlying PRP-hydrated particulated cartilage allograft. The autologous sealants had better delamination resistance than the allogeneic sealant.

**Clinical Relevance:**

The time-zero model is critical in elucidating the retention properties of fibrin and allogenic sealants after cartilage repair and before healing processes help stabilize the repair.

## Introduction

Fibrin sealants are used to supplement standard surgical techniques, such as suture or ligature, as an adjunct to hemostasis, or in cartilage repair surgeries to retain grafts or cells in a chondral defect.^1^^,^^2^ Sealants may be derived from autologous or commercial allogeneic sources and contain a fibrinogen and thrombin source. Upon activation via thrombin, fibrinogen is converted to fibrin consisting of a three-dimensional matrix of fibers.

Autologous fibrin sealants are derived from centrifuged whole blood components, such as platelet-rich plasma (PRP) or platelet-poor plasma (PPP) as a fibrinogen and thrombin source. This is contrary to allogeneic commercial fibrin sealants, such as TISSEEL (Baxter Healthcare Corporation, Deerfield, IL), that are developed from pooled human plasma. TISSEEL contains approximately 30-fold higher fibrinogen concentration than physiological levels present in autologous blood products.^3^^,^^4^ Higher fibrinogen concentration contributes to increased clot integrity; however, this may not benefit chondral repair due to inhibition of matrix synthesis and cell migration.^5^, ^6^, ^7^ Alternatively, using autologous PRP and PPP as a fibrin sealant provides a source of growth factors and physiological clot structure that could aid in tissue repair and regeneration,^5^ while potentially providing stability to the chondral graft.^2^

A previous study compared allogeneic and autologous fibrin sealants in a small-scale ex vivo cartilage repair model and demonstrated mechanical equivalence of autologous PPP-derived sealants and an allogeneic sealant.^2^ However, there are limited comparative biomechanical studies investigating full-scale chondral defect graft fixation ex vivo with controlled joint compressive load and anatomical shear motion representative of postoperative rehabilitation. The biomechanics study presented in this article addresses this limitation in literature, while comparing an allogeneic sealant to PRP- and PPP-derived sealants at time zero. The time-zero model is critical in elucidating the retention of the full-construct repair during the early postoperative rehabilitation, before healing processes help stabilize the repair.

The purpose of this study is to assess the integrity of chondral defect repairs filled with a cartilage allograft and sealed with either allogeneic fibrin sealant or autologous fibrin sealants created with PRP or PPP in a cadaver model. We hypothesized that the fibrin sealants would be comparable when analyzing graft fixation and have the same level of delamination from the underlying graft after testing.

## Methods

### Autologous Biologics Preparation

Institutional Review Board (IRB) approval was obtained before collecting human blood samples for the autologous preparations (Salus IRB 1082). Ninety cc of whole blood anticoagulated with anticoagulant citrate dextrose solution (ACD-A; Citra Labs, Braintree, MA, U.S.A.) to a final concentration of 13.3% (vol/vol) was collected from five human donors consisting of two males and three females with an average age of 26.0 ± 3.5 years. PRP and PPP fractions were prepared with the Arthrex Angel cPRP system (Arthrex, Naples, FL) set to 7% hematocrit. After centrifugation and automatic plasma separation by the machine, PRP was expanded to approximately 7 cc by pulling back on the PRP syringe until the desired volume was achieved. Complete blood counts (CBC) of whole blood, PRP, and PPP fractions were obtained with a Sysmex XE-5000 (Sysmex America, Lincolnshire, IL).

Autologous thrombin was prepared using the Thrombinator System (Arthrex, Naples, FL) and PPP. Initially, 0.1 cc CaCl_2_ (10%, 1.36 mEq; International Medication Systems, South El Monte, CA) and 4 cc PPP were injected into the device and allowed to clot for at least 15 minutes. When the defect was repaired and ready for fixation, the clot in the device was broken by shaking per the manufacturer’s instructions and an additional 0.2 cc CaCl_2_ and 8 cc PPP were injected into the Thrombinator device. One minute after clot reformation, the clot was shaken briefly, and the serum was extracted from the device through an 18-μm filter. A small sample of thrombin serum was used to evaluate comparative clotting time at a 1:1 ratio with a pooled fibrinogen source on a STart4 Hemostasis Analyzer (Diagnostica Stago, Parsippany, NJ).

### Defect Creation and Repair

Five, fresh-frozen human male cadaveric knee samples (Science Care, Phoenix, AZ) of age ≥55 years were used in this study. Before use, each sample was evaluated radiographically and arthroscopically via NanoScope operative arthroscopy imaging system (Arthrex, Naples, FL) to confirm there was no osteoarthritis exceeding Jäger-Wirth grade 2 on the distal MFC.

**Fig 1:**
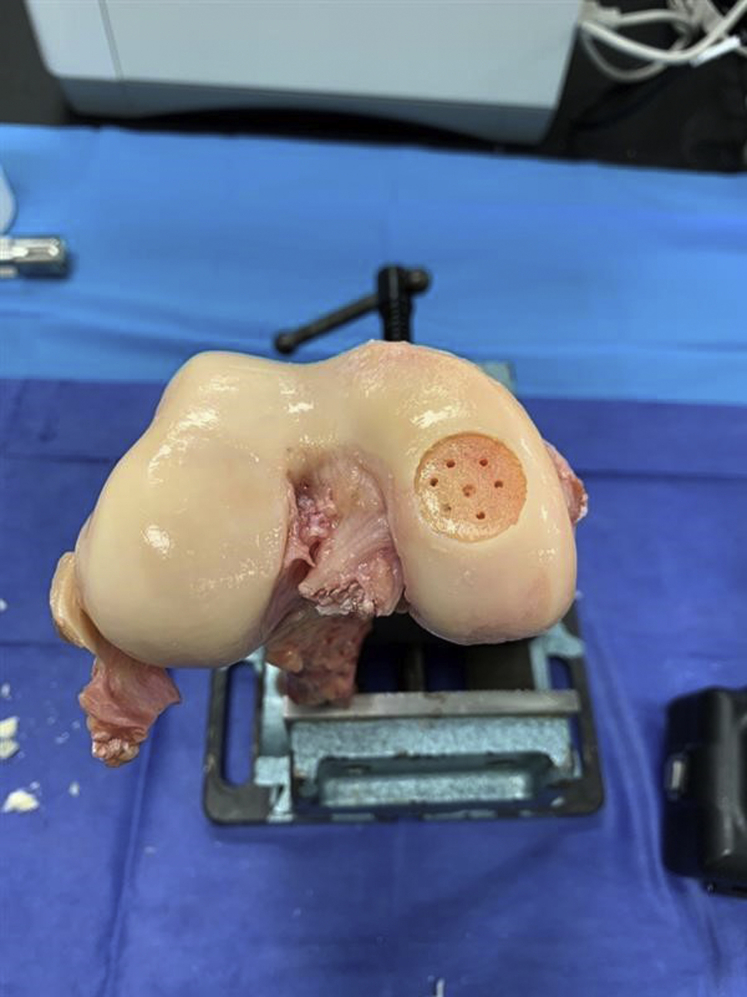
Right femur exhibiting a reamed 20-mm medial femoral condyle defect with removal of the calcified chondral layer and microdrilling performed using a 1.5-mm drill.

The isolated femur was positioned upright and gripped securely in a vise containing serrated jaws. Using an Allograft OATS Harvester with a depth stop device (Arthrex, Naples, FL), a-20 mm diameter defect was reamed normal to the distal medial articular surface to a depth of 1.5 mm. A ring curette was used to remove the calcified chondral layer and to establish the vertical defect walls. Microdrilling was then performed using a 1.5-mm, 45° PowerPick Drill, with a drill depth of 6 mm (Arthrex, Naples, FL). The defect was cleaned of debris before chondral defect repair (Fig 1). For all samples, BioCartilage allograft matrix (Arthrex, Naples, FL) was hydrated with PRP in a 1:1 ratio (0.5 cc:0.5 cc), and the defect was filled with the mixture until slightly recessed to approximately .5 mm.

**Fig 2:**
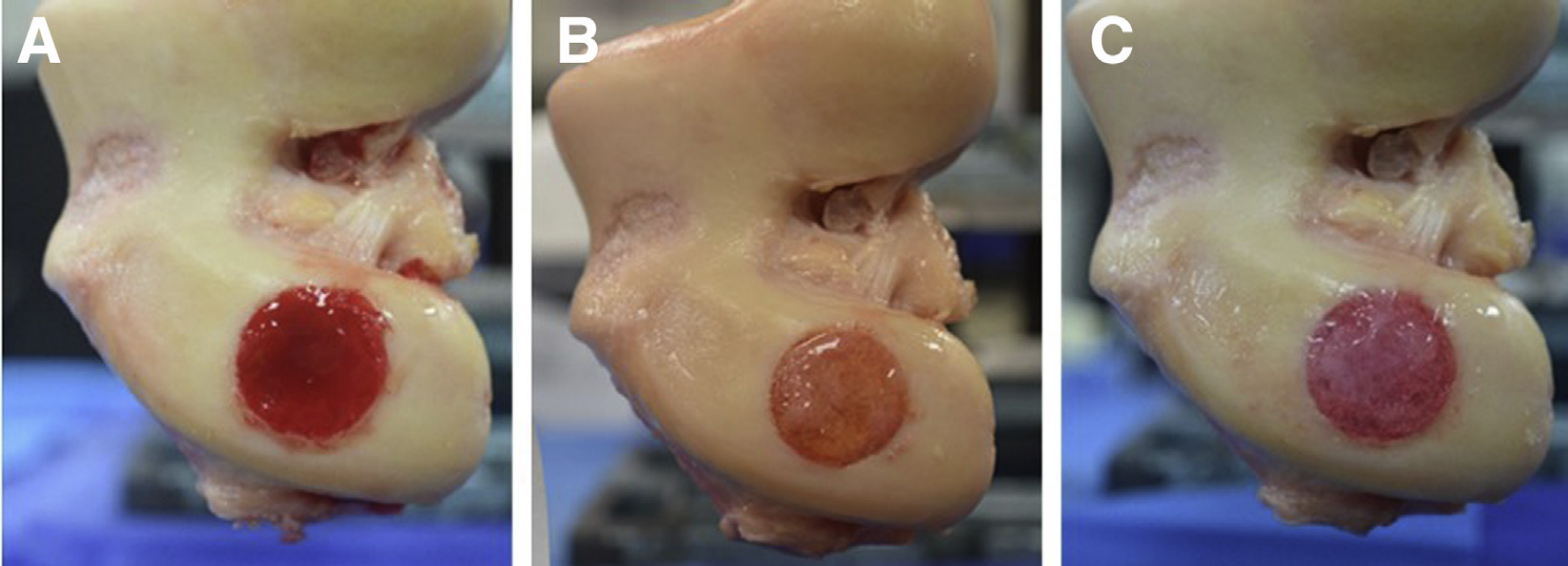
Each medial femoral condylar defect (left femur shown) was repaired by filling with a slightly recessed layer of a particulated cartilage matrix hydrated with platelet-rich plasma (PRP), followed by fibrin sealants created with PRP (A), platelet poor plasma (PPP) (B), or allogeneic (C) sealant.

Three fibrin sealant formulations were evaluated in this study: 1) PRP as a fibrinogen source with autologous thrombin, 2) PPP as a fibrinogen source with autologous thrombin, and 3) all-allogeneic fibrinogen and thrombin (TISSEEL, Baxter Healthcare Corporation, Deerfield, IL). Autologous thrombin (3 cc) was transferred to a 1:1 applicator assembly along with 3 cc of a fibrinogen source (PPP or PRP). A blending connector with mixer (Arthrex, Naples, FL) was used to mix and dispense the sealant over the defect until flush with the articular surface (Fig 2, A-C). The allogeneic sealant was thawed for at least 1 hour, but no longer than 4 hours, at 37°C per manufacturer instructions before application.

In this study, each femoral defect was repaired and tested three consecutive times using the different sealants in a randomized order. Before each repair, the defect was thoroughly irrigated with phosphate-buffered saline (PBS) 1× (VWR, Radnor, PA) to remove remnant particulated cartilage and sealant from the previous round, similar to Bogunovic et al.^8^ The autologous blood products of one blood donor were paired to one femur to eliminate cross-sample variability between human whole blood samples and cadaver femur samples.

### Biomechanical Testing

**Fig 3:**
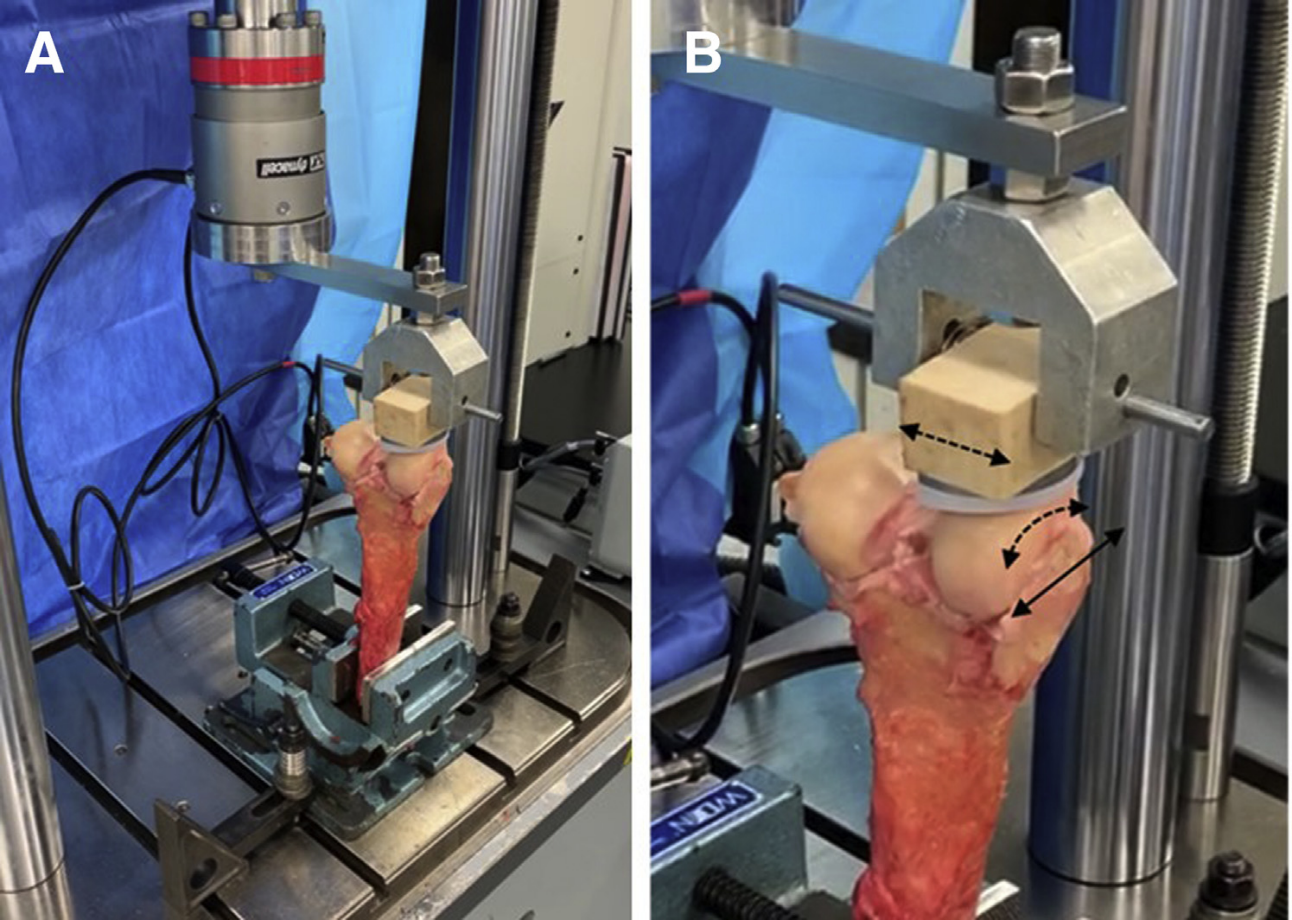
(A) The medial femoral condylar defect (right femur shown) was centered beneath a tibial bearing lubricated with bovine synovial fluid and compressively loaded to 44 N. The full repair was cycled 60 times to simulate shear motion encountered during continuous passive motion. (B) The tibial construct was designed with degrees of freedom (black dashed arrows) to maintain articulation with the femur while linearly displacing (solid black arrow) over the defect.

The MFC defect repairs were tested using an Instron ElectroPuls multiaxial mechanical testing system with a 1 kN/25 Nm load cell (Instron, Norwood, MA) to simulate the cyclic shear forces experienced by the repair during postoperative continuous passive motion (CPM). An articulating iBalance Unicondylar Knee Arthroplasty tibial bearing (Arthrex) made of ultra-high-molecular-weight-polyethylene (UHMWPE) was used in place of a cadaveric tibia for consistency between groups and to circumvent concerns about variable tibial osteoarthritis in cadavers. The tibial bearing was implanted into a 30 lb/ft^3^ foam block (Sawbones, Vashon Island, WA) using the appropriate instrumentation and polymethylmethacrylate (PMMA) cement (Benco Dental, Pittston, PA). A dowel pin was used to traverse the width of the foam block and hold it via a clevis, enabling a rotational degree of freedom and minor lateral degree of freedom for the tibial implant to linearly track the MFC surface while cycling and, thus, be quasi-physiological for the flexion-extension mechanism (Fig 3B).

The vise was positioned and secured on the Instron base plate, so that the defect was centered beneath the tibial bearing. Before articulation, both the tibial bearing and femoral condyle were lubricated with bovine synovial fluid (Lampire Biological Laboratories, Pipersville, PA) to replicate the in vivo environment. Using WaveMatrix software, a compressive load of 44 N, an estimated tibiofemoral joint reaction force during passive motion,^9^ was applied to the MFC repair over 10 seconds and then held for the duration of testing (Fig 3, A and B). Under rotary control, the tibial implant cycled over the full length of the repair at a rate of .036 rad/s for 60 cycles.

### Analysis of Defect Repair

**Fig 4:**
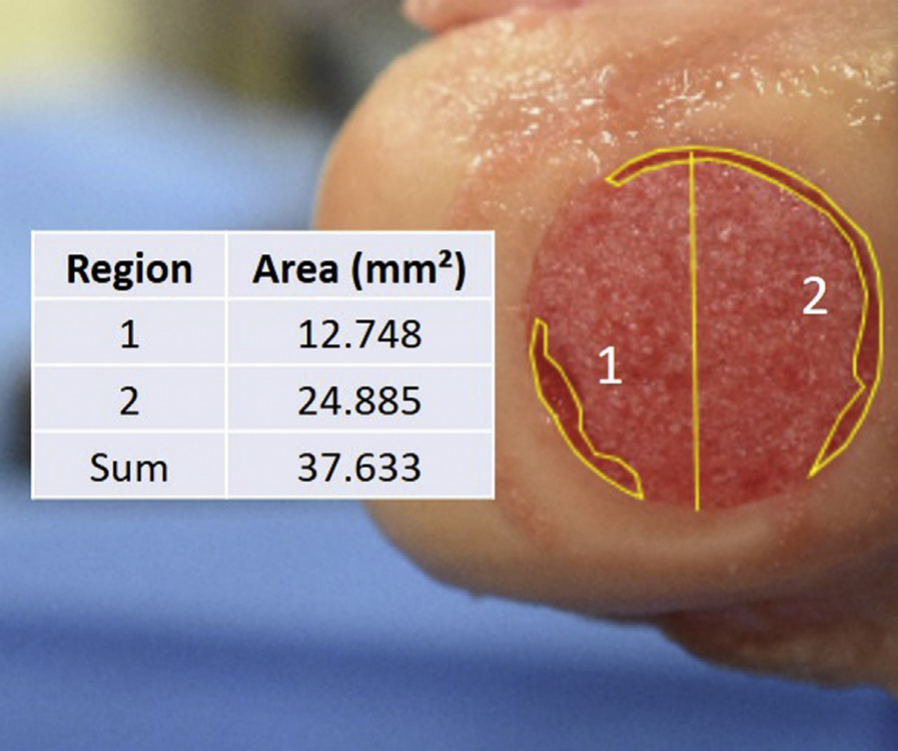
ImageJ was used to determine defect exposure areas by first scaling pixels to mm (center vertical line) and then creating and measuring regions of interest (regions 1 and 2). The summed areas were calculated as a percentage of the whole defect surface area for calculation of defect exposure.

Immediately before and after testing, images were taken with a digital camera (Nikon, Tokyo, Japan). The images after testing were analyzed with ImageJ software (National Institutes of Health, Bethesda, MD) to calculate the percentage of defect exposure. Defect exposure in this study was defined as visible loss or contractility of the particulated cartilage matrix (i.e., loss of repair concentricity) when compared to the pretest image. The scale was set by correlating pixels to the reamed 20-mm diameter of the defect. Using the polygon selection tool, the regions of loss were manually outlined and summed to determine the approximate area of defect exposure (Fig 4). The percentage of defect exposure was calculated by dividing over the whole defect area (314.16 mm^2^).

Comparable graft fixation was defined as having no observed significant difference between groups regarding defect exposure while also having significantly less than 30% defect exposure. The 30% value was calculated specifically for this study using the critical size defect of 6 mm, which is the defect dimension that is incapable of repairing without intervention, determined from well-accepted preclinical goat models of cartilage repair.^10^, ^11^, ^12^

After testing, the sealant delamination was also assessed by two unblinded orthopaedic surgeons (of whom one is fellowship-trained) on a scale of 0-5, similar to Bekkers et al.,^13^ where the scores reflect increasing delamination of 0%, 1-24%, 25-49%, 50-74%, 75-99%, and 100%, respectively. Sealant delamination was defined as the percent loss of the sealant area coverage over the underlying graft. To determine a qualitative assessment of sealant delamination, the mean value of the cumulative responses for each group was calculated and compared.

### Statistics

Statistical analysis was performed in SigmaPlot 14.0 (Systat, San Jose, CA) and Minitab 19 (State College, PA) with all statistics using α = .05 and β = .20. For parametric data, independent *t*-tests were used to evaluate any differences in cell content in PRP and PPP autologous fractions from the blood donors; if data were not normally distributed, the nonparametric Mann-Whitney rank sum test was used. Parametric independent *t*-tests were also used to compare clotting times of the autologous thrombin serum used with PRP and PPP sealants. A parametric one-way repeated measures analysis of variance was used to compare the defect exposures between the autologous and allogeneic sealant groups. Parametric one-sample *t*-tests were used to compare the mean defect exposures to the critical value of 30%. Interobserver reliability for sealant delamination was calculated via Kendall’s coefficient of concordance. The post hoc power analysis used a minimal statistical power of .80.

## Results

### Autologous Biologics Analysis

Complete blood counts (CBC) of the PRP and PPP fractions derived from each patient’s whole blood were analyzed for confirmation that the autologous fractions had significantly different compositions and were consistent with known expected values for PRP and PPP (Table 1). PRP had a significantly higher white blood cell count (17.60 ± 5.83 K/μL vs 0.04 ± 0.03 K/μL; *P* = .003), platelet count (1667.4 ± 427.9 K/μL vs 60.4 ± 20.7 K/μL; *P* = <.001), and red blood cell count (0.790 ± 0.076 M/μL vs 0.008 ± 0.005 M/μL; *P* = .008).

**Table 1:** Analysis of Mean Values of Autologous Biologics Components Prepared From Whole Blood of Human Donors

Sealant Fibrinogen Source	Thrombin Clot Times (s)	Blood Component Concentration		
		WBC (K/μl)	RBC (M/μl)	PLT (K/μl)
PPP (Means ± SD)	5.1 ± 0.6	0.04 ± 0.03	0.008 ± 0.005	60.4 ± 20.7
PRP (Means ± SD)	4.8 ± 0.4	17.60 ± 5.83	0.790 ± 0.076	1667.4 ± 427.9
*P* value	.47	.003††	.008†	<.001

PLT, platelets; PPP, platelet-poor plasma; PRP, platelet-rich plasma; RBC, red blood cells; WBC, white blood cells.

† Non-normal distribution; Mann Whitney Rank Sum test used.

†† Unequal variance; Welch’s *t*-test used.

The comparative clotting times between the thrombin serum used to clot either PPP or PRP fibrinogen sources were confirmed to not be significantly different (*P* = .47, Power = .104) as seen in Table 1.

### Graft Fixation and Delamination

The mean defect exposures calculated for different sealant fibrinogen sources were 4.20% ± 5.02% for PRP, 4.60% ± 5.18% for PPP, and 1.80% ± 2.95% for allogeneic sealant (Table 2). There were no significant differences observed for defect exposure between autologous and allogeneic sealants when used in conjunction with underlying particulated cartilage hydrated with PRP (*P* = .227, Power = .143). Each group had significantly less defect exposure when compared to the critical value of 30% (*P* = <.001 for all).

**Table 2:** Defect Exposure and Qualitative Assessment of Sealant Delamination After Testing

Sealant Fibrinogen Source	Defect Exposure (%)	Sealant Delamination (0-5)
PRP	4.20 ± 5.02	0.70 ± 0.67
PPP	4.60 ± 5.18	0.50 ± 0.53
Allogeneic	1.80 ± 2.95	1.30 ± 0.82

All values are reported as means ± SD. The sealant delamination scale of 0-5 reflects the increasing degree of delamination, where 0 is 0%, 1 is 1-24%, 2 is 25-49%, 3 is 50-74%, 4 is 75-99%, and 5 is 100%. PPP, platelet-poor plasma; PRP, platelet-rich plasma.

**Fig 5:**
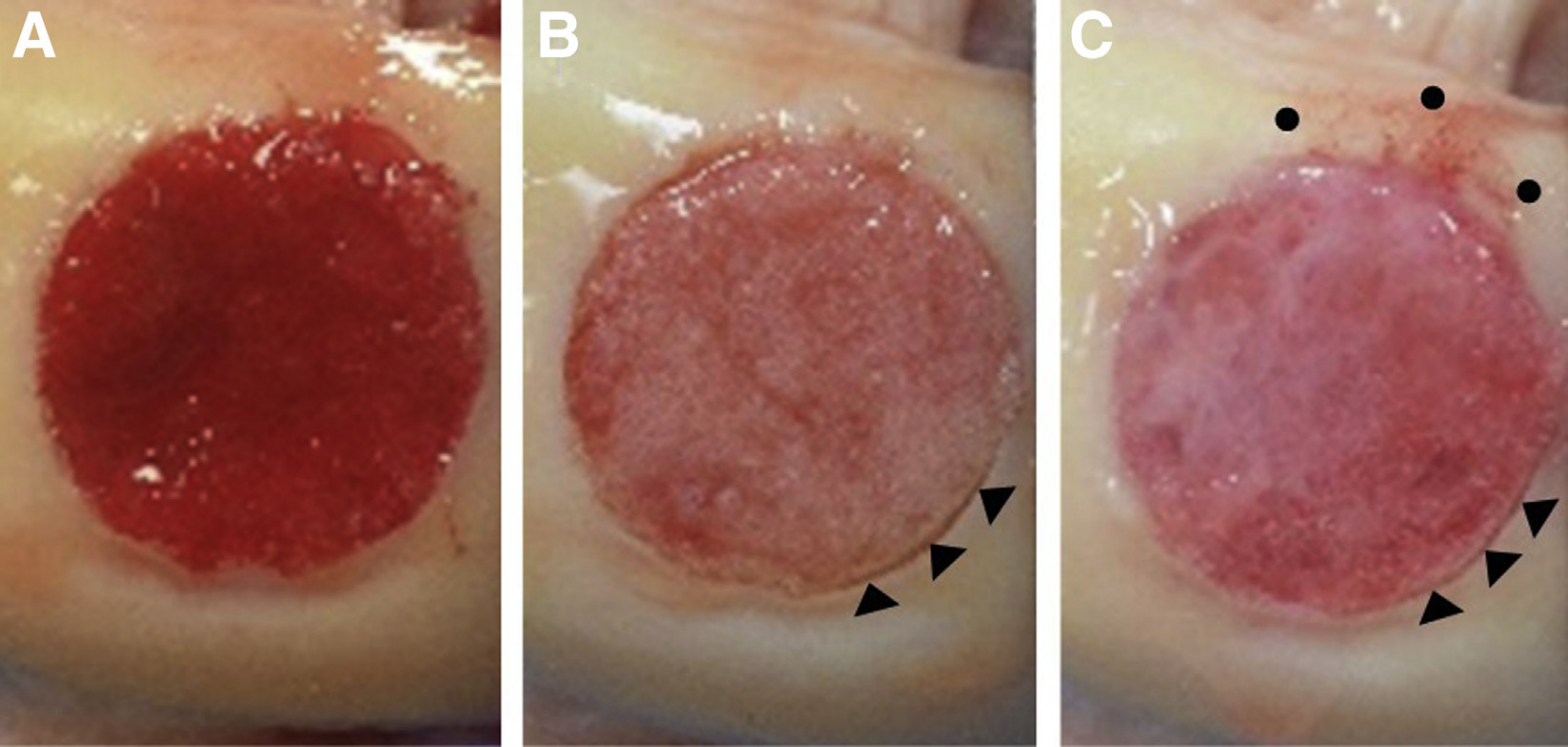
Sealants used in the same medial femoral condyle defect presented after testing. (A) Platelet-rich plasma (PRP) showed no defect exposure or sealant delamination. (B) Platelet-poor plasma (PPP) had minor defect exposure presenting as repair contractility (black triangles) without sealant delamination. (C) The allogeneic sealant had minor defect exposure presenting as repair contractility (black triangles) with some sealant delamination (black circles).

The sealants were analyzed qualitatively on a scale of 0-5 for sealant delamination as seen in Table 2 and Fig 5, A-C. The mean scores for PRP, PPP, and allogeneic sealant were 0.70 ± 0.67, 0.50 ± 0.53, and 1.30 ± 0.82, respectively. There were no observed instances of complete delamination. The Kendall’s Coefficient of Concordance was 0.708, indicating strong agreement between the two observers.

## Discussion

The main finding of this study is that no significant differences were observed between groups regarding defect exposure, and each group had significantly less defect exposure when compared to the critical value of 30%. No complete sealant delamination was observed after testing. However, the allogeneic sealant delaminated with a higher magnitude than did the autologous sealants. Thus, the hypothesis that the fibrin sealants would be comparable for graft fixation is accepted, while the hypothesis that the fibrin sealants would have the same level of delamination from the underlying graft is not accepted.

The ex vivo cyclic testing model controlled the applied compressive load based on physiological approximations of passive tibiofemoral joint reaction forces.^9^ A previous study evaluated micronized cartilage stability in the MFC by loading the quadriceps tendon of the wholly intact cadaver knee with a 10-lb weight and then performing 60 cycles of continuous passive motion.^8^ The limitation of that technique, which was addressed in the present experimental design, is the inability to quantify the compressive load applied to the defect due to a variety of cadaver-dependent features. The present study design overcomes this limitation by controlling the compressive load applied to each sealant during the shearing motion. This study was further controlled by testing each sealant in the same femur to reduce cross-sample variability and using an anatomically contoured, burr-free UHMWPE tibial bearing, instead of a highly variable cadaveric tibia.

A previous in vitro cartilage explant study evaluated the mechanical properties of PRP, PPP, and allogeneic fibrin sealants used in conjunction with an underlying particulated cartilage allograft. The authors found no difference in bulk failure properties or microscale strains at the graft-cartilage interface between the PPP and allogeneic sealants. PRP sealants, however, were determined to be inferior because of their higher observed strains through the graft-cartilage interface depth.^2^ The present study did not observe significant differences in defect exposure between PRP, PPP, and allogeneic sealants, which may infer that the higher microscale strains of PRP constructs are inconsequential to graft retention during early postoperative rehabilitation.

A theory for the sealant displacement observations is derivative of that proposed by Irwin et al.^2^ Allogeneic sealants like TISSEEL, which have substantially higher fibrinogen concentration and thrombin activity compared to the autologous sealants,^4^^,^^14^ instantly generate a clot. Consequently, it forms a distinct layer atop the particulated cartilage matrix. whereas the autologous sealant groups have several seconds to diffuse into the underlying matrix while clotting. When subjected to compression and shear forces, the allogeneic sealant layer is, thus, more likely to separately displace due to its lesser integration into the graft.

### Clinical Impact

The allogeneic sealant TISSEEL contains supra-physiological levels of fibrinogen with reported levels between 67 and 106 mg/ml. In contrast, both PRP and PPP have much lower fibrinogen concentrations that range from 2 to 4 mg/ml.^2^, ^3^, ^4^ Even with these lower fibrinogen levels, robust clot formation was still observed with the autologous products and the autologous and allogeneic sealants had similar initial mechanical integrity when used in MFC chondral defect repairs.

While unable to be evaluated as part of this study, there is evidence that autologous formulations may augment repairs from a biological perspective. For example, allogeneic sealants with supraphysiological fibrinogen concentration have been shown to create a dense barrier that hinders necessary functions in wound healing, such as cell migration and tissue formation.^15^^,^^16^ Allogeneic sealants may also contain supraphysiological thrombin activity relative to autologous preparations (400-625 U/ml^4^ vs 10-15 U/ml^14^). In a wound-healing model, wounds treated with a fibrin matrix containing lower thrombin concentrations (4 IU/ml) appeared less severe and displayed more rapid wound healing than wounds treated with fibrin matrix containing higher thrombin concentrations (800 IU/ml).^17^

Autologous sealants may also serve as a vector for enriched growth factor delivery within the particulated cartilage graft, which also contains bioactive factors supporting cell growth and chondrogenic differentiation.^18^ PRP contains concentrated platelets that, upon activation with thrombin, release growth factors, such as platelet-derived growth factor, transforming growth factor (TGF)-β1 and TGF-β2, which are beneficial for wound healing.^19^^,^^20^ PPP also contains plasma proteins that are advantageous in cartilage repair, such as insulin-like growth factor.^21^ As demonstrated herein, autologous sealants can be varied with either fibrinogen or thrombin source without impacting initial defect stability, while having different concentrations of platelets, leukocytes, and growth factors.^3^

The allogeneic sealant investigated in this study (TISSEEL) contains thrombin and fibrinogen developed from pooled human plasma, as opposed to an all-autologous fibrin sealant. Although vapor heat and solvent detergent treatments are performed for viral reduction, no procedure has been proven to eliminate viral contamination, posing the risk of infectious transmission and immunogenic response.^5^ Unlike autologous sealants, TISSEEL contains aprotinin, an antifibrinolytic protease serving to increase the resistance of the clot to degradation, which can cause anaphylactic reactions.^4^ Whole blood collected from heparinized patients or patients with coagulopathy cannot produce a PRP or PPP product that is capable of clotting, indicating autologous fibrin treatments would not be an option in these situations.

### Limitations

This study is not without limitations. One limitation of this model is that fibrin sealants were subjected to cyclic loading within 5 minutes following clot formation without immobilization to allow for repair stabilization before loading as is standard in clinical practice.^22^ Ex vivo conditions prohibit the recommended defect immobilization during postoperative healing. Moreover, our study used cadaveric knee specimens, which eliminates the benefit of active bone marrow stimulation during microdrilling that could aid in clotting and construct retainment. As a result, the in vivo performance of the repairs cannot be fully determined from this study; however, the study conditions may represent a worst-case scenario as no healing had occurred to further stabilize the repair. The effects of other compressive loads, fluid environments, shear rates, and defect filling (i.e., proud vs recessed) were not evaluated in this study, but they may influence the outcomes presented in this study. Testing was also performed at room temperature as opposed to physiological temperature, although this did not impact the ability of the sealants to form and maintain a robust clot.

Furthermore, the sample size was small; we are unable to rule out beta-error. A post hoc power analysis revealed that 72 specimens would be needed to achieve a power of .80. Because of considerations surrounding the use of cadavers, the study was not expanded further for increased power. Additionally, intraobserver reliability was not performed for the sealant delamination analysis. The surgeons were also not blinded, as color differences were required to observe regions of delamination.

## Conclusions

The PRP and PPP sealants were comparable to the allogeneic sealant for graft fixation when used in conjunction with an underlying PRP-hydrated particulated cartilage allograft. The autologous sealants had better delamination resistance than the allogeneic sealant.

## Supplementary Material

Full ICMJE author disclosure forms
